# The Expression Patterns and Implications of *MALAT1*, *MANCR*, *PSMA3-AS1* and *miR-101* in Esophageal Adenocarcinoma

**DOI:** 10.3390/ijms25010098

**Published:** 2023-12-20

**Authors:** Athanasios Syllaios, Maria Gazouli, Michail Vailas, Konstantinos S. Mylonas, Stratigoula Sakellariou, Stavros Sougioultzis, Ioannis Karavokyros, Theodoros Liakakos, Dimitrios Schizas

**Affiliations:** 1 First Department of Surgery, Laikon General Hospital, National and Kapodistrian University of Athens, 115 27 Athens, Greece; mike_vailas@yahoo.com (M.V.); iokaravokyros@msn.com (I.K.); theodlia@hotmail.com (T.L.); schizasad@gmail.com (D.S.); 2 Laboratory of Biology, Department of Basic Medical Sciences, Medical School, National and Kapodistrian University of Athens, 115 27 Athens, Greece; mgazouli@med.uoa.gr; 3 Department of Cardiac Surgery, Onassis Cardiac Surgery Center, 176 71 Athens, Greece; ksmylonas@gmail.com; 4 First Department of Pathology, Medical School, National and Kapodistrian University of Athens, 115 27 Athens, Greece; sakellarioustrat@yahoo.gr; 5 Gastroenterology Unit, Department of Pathophysiology, School of Medicine, National and Kapodistrian University Athens, 115 27 Athens, Greece; ssougioultzis@yahoo.com

**Keywords:** LncRNA, miRNA, MALAT1, MANCR, PSMA3-AS1, miR-101, esophageal cancer, adenocarcinoma

## Abstract

Esophageal adenocarcinoma (EAC) is a malignant tumor with poorly understood molecular mechanisms. This study endeavors to elucidate how the long non-coding RNAs (lncRNAs) *MALAT1*, *MANCR* and *PSMA3-AS1*, as well as the microRNA *miR-101*, exhibit specific expression patterns in the pathogenesis and prognosis of EAC. A total of 50 EAC tissue samples (tumors and lymph nodes) and a control group comprising 26 healthy individuals were recruited. The samples underwent quantitative reverse transcription-polymerase chain reaction (qRT-PCR) analyses. The relative expression levels of MALAT1, MANCR, PSMA3-AS1, and miR-101 were ascertained and correlated with various clinicopathological parameters including TNM staging, tumor characteristics (size and grade of the tumor) lymphatic invasion, disease-free (DFS) and overall survival (OS) of EAC patients. Quantitative analyses revealed that MALAT1 and MANCR were significantly upregulated in EAC tumors and positive lymph nodes when compared to control tissues (*p* < 0.05). Such dysregulations correlated positively with advanced lymphatic metastases and a higher N stage. DFS in the subgroup of patients with negative lymph nodes was higher in the setting of low-MANCR-expression patients compared to patients with high MANCR expression (*p* = 0.02). Conversely, miR-101 displayed a significant downregulation in EAC tumors and positive lymph nodes (*p* < 0.05), and correlated negatively with advanced tumor stage, lymphatic invasion and the grade of the tumor (*p* = 0.006). Also, patients with low miR-101 expression showed a tendency towards inferior overall survival. PSMA3-AS1 did not demonstrate statistically significant alterations (*p* > 0.05). This study reveals MALAT1, MANCR, and miR-101 as putative molecular markers for prognostic evaluation in EAC and suggests their involvement in EAC progression.

## 1. Introduction

The transcriptional activity of eukaryotic genomes encompasses approximately 90% of the genomic DNA. Merely 2% of these transcribed sequences encode proteins, with the majority constituting non-coding RNAs (ncRNAs). Depending on their size, ncRNAs consist of long ncRNAs (lncRNAs), small microRNAs (miRNAs), interfering RNAs (siRNAs) and PIWI-interacting RNAs (piRNAs) [1].

Non-coding RNAs (ncRNAs) play a crucial role in the human genome. Long non-coding RNAs (lncRNAs) are large molecules with varied functions, including chromatin remodeling, gene expression regulation, and carcinogenesis. Initially considered as “transcriptional noise”, lncRNAs have since been recognized for their significance [2,3,4]. Examples of upregulated cancer-associated lncRNAs currently studied in esophageal adenocarcinoma (EAC) include AL009178.2, AL135924.2, AL138789.1, *AC007128.1*, *AC079354.3*, *AP003356.1*, *AP0033469.2*, *GK-IT1*, *HOTAIR*, *LINC01114*, *LINC01768*, *LINC01612*, *AC008443.2*, and *LINC02582*, all of which have been correlated with poor prognosis when overexpressed [5,6]. Long intergenic noncoding RNAs (lincRNAs) represent a distinct evolutionary category within the broader class of lncRNAs, increasingly recognized for their regulatory functions in the progression of cancer. These molecules are also being investigated for their potential utility as biomarkers in cancer. Current research suggests that the upregulation of *lincPRKD* and *lincRTL* may serve as a valuable indicator for the early detection and therapeutic intervention of EAC [7]. On the contrary, microRNAs (miRNAs) are small ncRNAs that regulate gene expression. Dysregulated miRNAs are associated with various cancer-related processes, including cell proliferation, metastasis, and angiogenesis [8]. Examples of miRNAs investigated in the context of EAC and identified as exhibiting downregulation include miR-148a, miR-145, miR-383, miR-301b and miR-372 [5]. Furthermore, miRNAs have the capacity to interact with lncRNAs, influencing each other’s expression in a mechanism known as competing endogenous RNAs (ceRNAs) [9]. This initial ceRNA hypothesis has undergone thorough investigation, revealing that non-coding pseudogenes, as well as 3’ untranslated regions (3’UTR) or splicing variants of certain transcripts (such as the urokinase receptor), can function as molecular sponges. These entities engage in competitive activities, leading to the sequestration of oncosuppressor microRNAs and influencing the expression of their respective targets [10]. Both lncRNAs and miRNAs have emerged as key players in understanding gene regulation and cancer biology.

Metastasis-associated in lung adenocarcinoma transcript 1 (MALAT1), mitotically associated long non-coding RNA (MANCR) and proteasome subunit α3 antisense RNA 1 (PSMA3-AS1) are long non-coding RNAs (lncRNAs) that have been implicated in various aspects of carcinogenesis, including tumor growth, metastasis, and drug resistance [8,9,11,12,13]. MALAT1 is a lncRNA that inhibits cell apoptosis and promotes invasion and migration in cancer [9]. Upregulation of MALAT1 negatively impacts prognosis in different types of cancers, including pancreatic cancer, hepatocellular carcinoma, nasopharyngeal carcinoma, colorectal cancer, breast cancer, osteosarcoma, gastric cancer (GC), lung cancer and esophageal squamous cell carcinoma (ESCC) [9]. MANCR has been studied extensively in breast cancer, as well as in head and neck squamous cell carcinoma, thyroid cancer, prostate cancer, lung cancer, lymphoma, gastric cancer and ESCC. In these settings, MANCR has been found to promote tumor growth, cancer cell proliferation, migration and invasion, leading to worse tumor characteristics and poor patient outcomes [11,14,15].

Similarly, *PSMA3-AS1* has been linked to increased cellular proliferation, migration, invasion and metastasis in multiple malignancies including cholangiocarcinoma, colorectal cancer, prostate cancer, lung cancer, multiple myeloma, ESCC, ovarian cancer and lymphoma [12,16]. Indeed, targeting these lncRNAs could potentially revolutionize the care of multiple malignancies.

**Figure 1:**
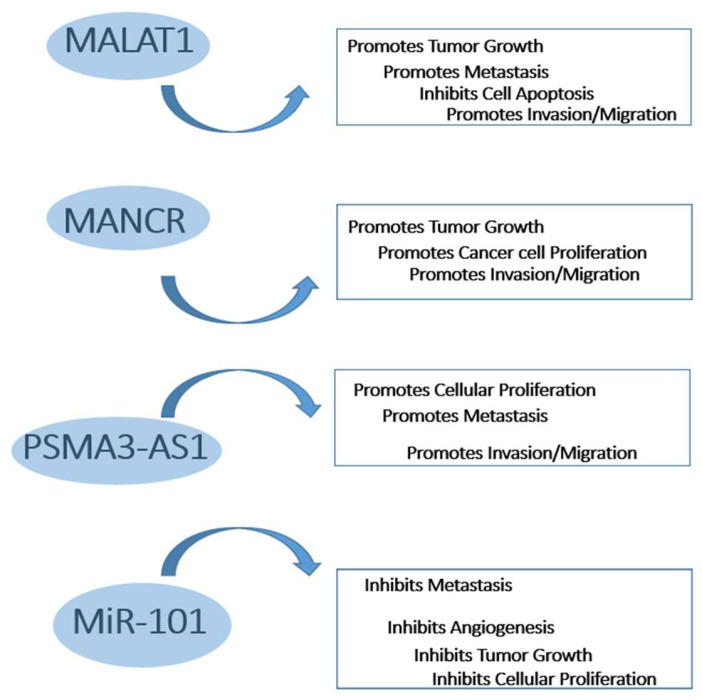
Schematic representation of possible mechanisms of actions of the studied molecules in cancer development.

On the other hand, *miRNA-101* (miR-101) is a tumor suppressor miRNA that inhibits the expression of cardinal oncogenes which participate in cell proliferation, survival, metastasis, and angiogenesis [17]. The downregulation of *miR-101* occurs through genetic and epigenetic mechanisms. Decreased miR-101 levels are associated with cancer development, progression, and poor prognosis in lung, breast, prostate, liver, bladder, gastric and colorectal cancer [17]. Of note, miR-101 has been found to serve as a possible ceRNA to *MALAT1*, *MANCR* and *PSMA3-AS1* [9,12,17]. Figure 1 illustrates some possible mechanisms of action of the studied molecules in cancer development.

Ultimately, targeting key lncRNAs, as well as restoring or enhancing *miR-101* expression, could potentially revolutionize the care of multiple malignancies [17].

The present study sought to identify expression patterns and possible clinical implications of those molecules in EAC patients. Although the aforementioned molecules have been studied in ESCC or gastric adenocarcinoma, their impact in EAC patient outcomes remain poorly defined.

## 2. Results

### 2.1. Demographics and Staging

Table 1 displays the demographic and clinical characteristics of both patients and the control group. Overall, 50 EAC patients and 26 health individuals were recruited. A male predominance was observed in both groups. Nineteen patients were grouped into stages T1 and T2, whereas thirty-one patients were grouped into stages T3 and T4. Twenty-nine patients were grouped into Grade 1 and 2 of the disease, whereas there were twenty-three Grade 3 patients. Eighteen patients were assigned to N0. The mean follow up was 48 months (range 3–60 months).

### 2.2. MALAT1, MANCR, PSMA3-AS1 and miR-101 Levels

#### 2.2.1. Expression Patterns of MALAT1, MANCR, PSMA3-AS1 and miR-101 Levels in EAC Tumors and Lymph Nodes

To investigate the expression of the studied molecules in EAC patients, tissue samples of EAC and matched adjacent non-cancerous tissues were detected by means of qRT-PCR and compared to healthy esophageal tissues. Positive lymph node tissues were also analyzed and compared to healthy tissues. *MALAT1* expression levels were notably higher in cancer tissues and positive lymph nodes compared with those of healthy controls (1.9 and 1.97 times higher than the controls, respectively, *p* < 0.05). Statistically significant upregulation of *MANCR* was observed in both EAC tissues and positive lymph nodes when compared to the controls, demonstrating increases of 2.58 and 2.11 times, respectively (*p* < 0.05). Conversely, miR-101 exhibited downregulation in EAC tumors and positive lymph nodes as compared to controls, showing reductions of 15.95 and 7.82 times, respectively, with statistical significance (*p* < 0.05). However, there was no statistically significant difference observed in the expression of PSMA3-AS1 between EAC tumors or positive lymph nodes and their corresponding healthy tissues. The aforementioned findings are summarized in Table 2. These results demonstrate that upregulation of *MALAT1*, *MANCR* and downregulation of *miR-101* are associated with the malignant development of EAC tumors. High expression levels of *MALAT1*, *MANCR* and low expression of *miR-101* are also closely associated with lymph node metastases/invasion.

#### 2.2.2. Dysregulation of MALAT1, MANCR, and miR-101 May Have an Impact on the Staging of EAC Tumors

**Figure 2:**
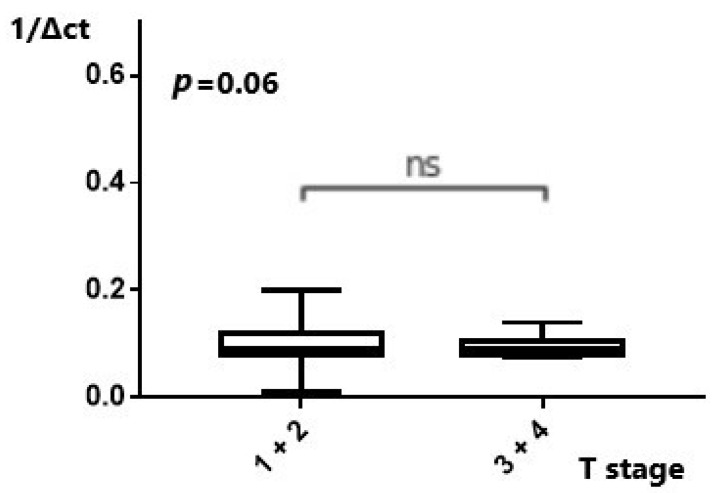
Expression of Mir-101 in relation to grouped T stage. Downregulation of Mir-101 was found in advanced T stages. ns: not significant.

We further tried to investigate whether dysregulation of the studied molecules resulted in higher EAC tumor stages (T stage and N stage). As regards the T stage, comparison of the *MALAT1* expression between low T and high T stages of disease did not reveal a statistically significant difference (*p* = 0.58) (Supplementary Figure S1). Analysis of *MANCR* expression in relation to the T stage also did not produce a statistically significant result (*p* = 0.07) (Supplementary Figure S2). The comparison of PSMA3-AS1 concentrations between the early (low T stage) and advanced (high T stage) disease states did not yield statistically significant differences. (*p* = 0.54) (Supplementary Figure S3). In *miR-101*, no statistically significant correlation, but rather a tendency, was found when pooled results of the T1/2 stages were compared with the results of the T3/4 stages, (*p* = 0.06) (Figure 2). Downregulation of *miR-101* resulted in higher T stages.

**Figure 3:**
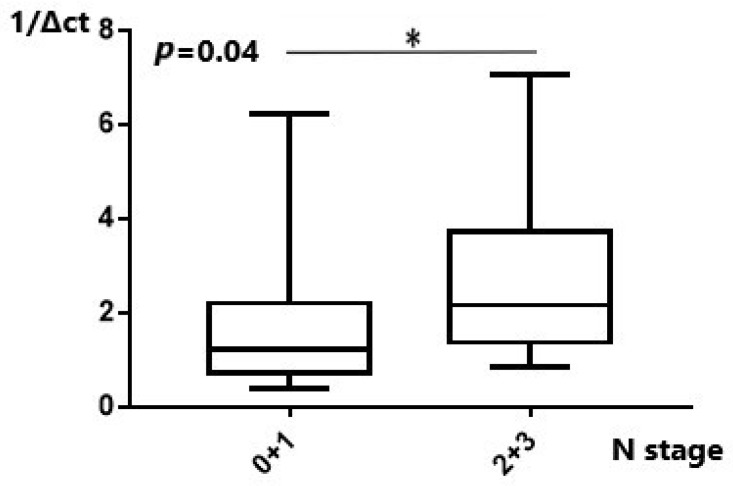
Expression of MALAT1 in relation to N stage. Upregulation of MALAT1 was found in advanced N stages. *: *p* < 0.05.

**Figure 4:**
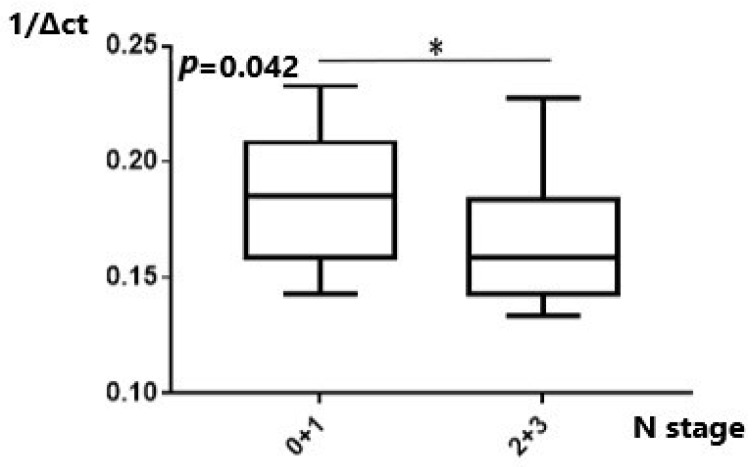
Expression of *MANCR* in relation to N stage. Lower MANCR expression was found in higher N stages. *: *p* < 0.05.

As regards the N stage, *MALAT1* was significantly upregulated in patients with N2 disease compared to N0 patients (Table 3). When the results for N0 and N1 patients were grouped and compared to the pooled results of groups N2 and N3, statistical significance was also achieved (*p* = 0.04) (Figure 3). *MANCR* was significantly downregulated in the N2/N3 cohort group compared to N0/N1 patients. (Table 4). Lower *MANCR* expression was apparent in higher N stage disease (*p* = 0.042) (Figure 4). Analyzing the PSMA3-AS1 expression levels among different N stages and combining the results of N0/1 patients and N2/3 patients did not result in statistically significant findings (Supplementary Figure S4). Similarly, *miR-101* comparison of pooled N0/1 with the N2/3 groups was also statistically insignificant (*p* = 0.67) (Supplementary Figure S5).

#### 2.2.3. MiR-101 and Potentially PSMA3-AS1 Could Affect EAC Tumor Characteristics

**Figure 5:**
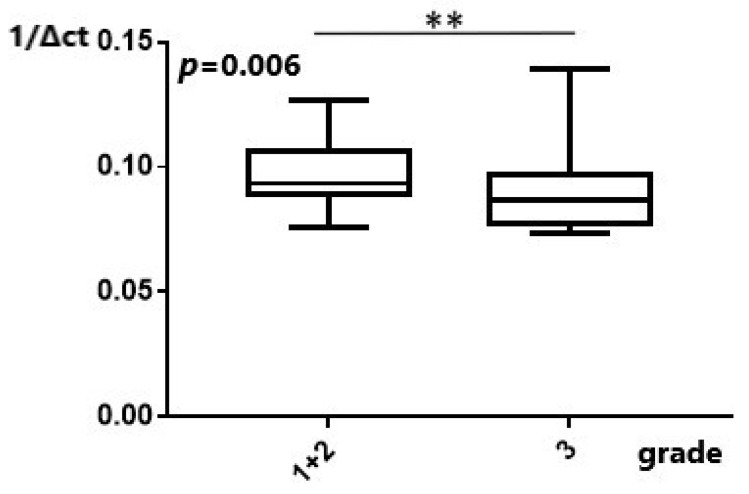
Expression of *miR-101* in relation to grade. Downregulation of *miR-101* is found in grade 3 tumors. **: *p* < 0.01.

**Figure 6:**
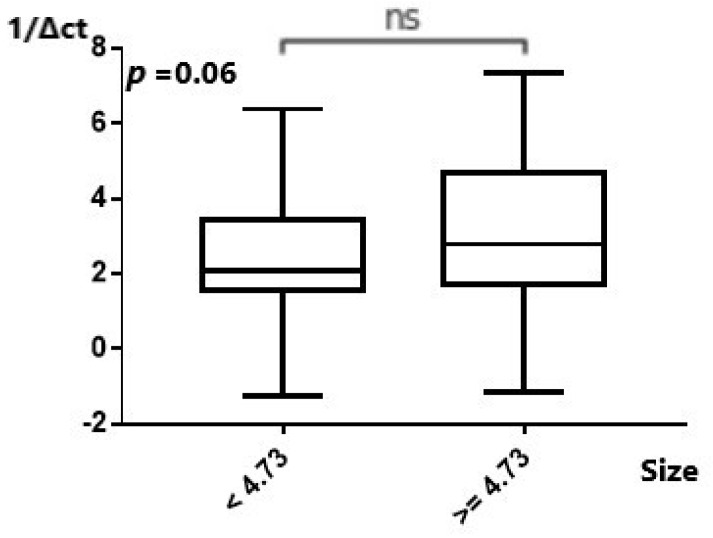
Expression of PSMA3-AS1 in relation to the size of the tumor. Higher PSMA3-AS1 expression in bigger tumors was observed. ns: not significant.

After this, we tried to investigate whether dysregulation of the studied molecules affect different EAC tumor characteristics (grade and size of the tumor). *MiR-101* expression was significantly higher in grade 1 and 2 tumors as opposed to grade 3 EAC tumors (*p* = 0.006) (Figure 5). Assessment of the expression of *miR-101* in relation to the size of the tumor did not yield a statistically significant result (*p* = 0.63) (Supplementary Figure S6). Expression of *MALAT1*, in relation to the size of the tumor, was not statistically significant either (*p* = 0.13) (Supplementary Figure S7). Similarly, there was no statistically significant difference in low and high tumor grades regarding *MALAT1* expression (*p* = 0.55) (Supplementary Figure S8). Assessment of *MANCR* expression in relation to grade and size of the tumor did not produce a statistically significant result (*p* = 0.07 and *p* = 0.21, respectively) (Supplementary Figure S9). Although the expression of *PSMA3-AS1* in relation to the size of the tumor was not statistically significant, a positive trend towards higher *PSMA3-AS1* expression in bigger tumors was demonstrated (*p* = 0.06), (Figure 6). No statistical significance in expression of PSMA3-AS1 was apparent between patients with grade 1 and 2 disease when compared to patients with stage 3 EAC (*p* = 0.45) (Supplementary Figure S10).

#### 2.2.4. MANCR and miR-101 Levels May Be Potential Prognostic Indicators for EAC Patients

**Figure 7:**
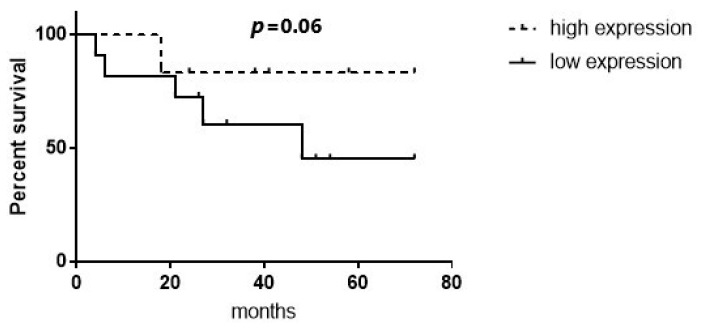
*miR-101*, overall survival (continuous line: low *miR-101* expression, intermittent line: high *miR-101* expression).

**Figure 8:**
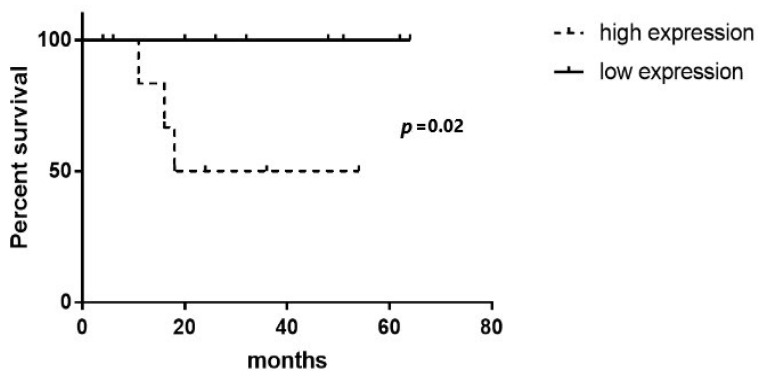
*MANCR*, disease-free survival in negative lymph-node patients (continuous line: low *MANCR* expression; intermittent line: high *MANCR* expression).

Finally, we investigated the disparities in overall survival (OS) and disease-free survival (DFS) between groups exhibiting low and high expression levels of the examined molecules, as well as in patients with negative lymph nodes. As regards *MALAT1*, OS and DFS did not significantly differ between the high-*MALAT1*- and low-*MALAT-1*-expression groups, when the mean concentration was 1/dct = 0.096 (high-MALAT1 > 0.096, low-MALAT1 < 0.096) (*p* = 0.09 and 0.76, respectively) (Supplementary Figure S11). On the other hand, patients with low *miR-101* expression showed a tendency towards inferior overall survival (*p* = 0.06) (Figure 7). Likewise, no statistically significant difference was observed in OS and DFS between the patient specimens exhibiting high miR-101 expression and those displaying low miR-101 expression when considering cases with negative lymph nodes (*p* = 0.38 and 0.93, respectively) (Supplementary Figure S12). *MANCR* levels also did not influence OS and DFS (*p* = 0.45 and *p* = 0.07, respectively). A tendency was only observed in patients with high *MANCR* expression for poorer DFS (Supplementary Figure S13). DFS in the subgroup of patients with negative lymph nodes was higher in the setting of low-*MANCR*-expression patients compared to patients with high *MANCR* expression (mean value of 1/dct = 0.143) (*p* = 0.02) (Figure 8). *PSMA3-AS1* expression did not affect OS/DFS in EAC patients, irrespective of nodal status.

## 3. Discussion

*MALAT1* has already been investigated for its activity as an oncogene in various types of cancer, such as breast, endometrial, lung, gastric, ovarian, prostate, and thyroid cancer, along with other cancers [13,18,19]. *MALAT1* promotes carcinogenesis in many ways. First, in the context of cancer metastasis, *MALAT1* appears to instigate epithelial-to-mesenchymal transition (EMT) and enhance the metastatic potential of cancer cells through modulation of the EZH2-Notch1 signaling pathway. Notably, *MALAT1* functions as a ceRNA, exerting control over the expression of ZEB1 and ZEB2 by sequestering *miR-200a*. This action promotes the invasiveness and migratory capabilities of cancer cells by inducing EMT [20]. Conversely, the suppression of *MALAT1* results in the downregulation of stem cell-associated genes OCT4 and NANOG, leading to the inhibition of cancer cell proliferation and migration and the formation of tumor spheres, while simultaneously promoting cell apoptosis. The expression of β-catenin, Lin28, and EZH2 genes is also decreased upon *MALAT1* downregulation [21]. Recent studies reported a positive association between the expression of methyltransferase-like 3 (METTL3) and *MALAT1* in osteosarcoma patients. METTL3 is implicated in facilitating m6A modification of *MALAT1*, thereby amplifying the oncogenic functions and stability mediated by *MALAT1* [22]. Additionally, a study by Yadav et al. demonstrated that the elimination of MALAT1 enhances sensitivity to PARP inhibition by disrupting homologous recombination in prostate cancer patients. This suggests a potential alternative therapeutic strategy for individuals with castration-resistant prostate cancer [23].

In a manner consistent with our findings in EAC, elevated *MALAT1* expression is associated with malignant potential and lymphatic invasion across a diverse spectrum of other solid organ malignancies [9,24,25,26]. Although statistically significant associations were not observed herein, its upregulation has been implicated with advanced TNM staging, lymphatic invasion, extensive primary tumor size, poor tumor differentiation and distant metastasis in tumors [9]. Furthermore, inferior overall survival and disease-free survival of patients exhibiting high *MALAT1* expression have been reported in numerous types of cancer, such as ESCC, gastric cancer, prostate and breast cancer [9,24,27]. Our results correspond to the current published literature for other cancers. MALAT1 was overexpressed in EAC tumors compared to non-cancerous tissue, and its upregulation was associated with lymphatic metastases and advanced N stage. On the other hand, no correlation was observed between MALAT1 expression and tumor size or differentiation. Although there was a trend towards inferior OS in patients with diminished MALAT1, this failed to reach statistical significance. The lack of statistical significance in this finding may be attributed to the limited number of patients included in our study cohort.

MANCR favors carcinogenesis in a multifactorial fashion. First, it promotes cancer cell proliferation and progression by downregulating miR-122a and PDE4D [28,29]. *MANCR* may also serve as a pivotal target for the bromodomain and extra-terminal (BET) protein BRD4, exerting a crucial influence on cellular migration and the invasive properties of cancer [15]. MANCR is upregulated in cancerous tissues compared with their non-cancerous counterparts [11]. Fan J. et al. also reported that *MANCR* is upregulated in ESCC cases [29]. However, the investigation of MANCR expression in EAC tumors has not yet been addressed in the existing scientific literature. Our study supports the suggestion that *MANCR* is upregulated in EAC patients, which is compatible with the findings in other cancer types. Differences in *MANCR* expression have not been extensively studied in cancer as regards their role in tumor staging and tumor characteristics. In our study, we found that *MANCR* expression could affect the N stage of EAC patients. Furthermore, Yao L. et al. reported that upregulation of *MANCR* is indicative of an unfavorable prognosis in gastric cancer patients [11]. Despite the small number of N0 patients (n = 18) in our study, our results support the suggestion that a high MANCR expression predicts poorer DFS in these patients too.

*PSMA3-AS1* is a lncRNA that acts as an oncogene. Various pathways have been implicated in cancer tumorigenesis, cancer cell proliferation, migration and invasion, including regulation of the miR-329-3p/ALDOA axis, modulation of the miR-101/EZH2 axis, modulation of the miR-411-3p/HOXA10 pathway, and others [12,30,31]. In ESCC cases, it is known that *PSMA3-AS1* is upregulated. Moreover, increased *PSMA3-AS1* expression is positively correlated with larger tumor sizes and poorer prognosis [12]. On the other hand, our results demonstrate that *PSMA3-AS1* expression is not altered in EAC patients. *PSMA3-AS1* expression does not seem to significantly affect the course of EAC patients either. These results could be attributed to different pathways implicated with *PSMA3-AS1* in ESCC and EAC tumors.

Finally, *miR-101* functions as a suppressor in the process of carcinogenesis. The detailed mechanisms governing the impacts of miR-101 and the precise implications of miR-101’s dysregulation in EAC tumors remain incompletely understood to date [17]. Various mechanisms have been proposed to elucidate how the expression of miR-101 may impede the growth, proliferation, migration, invasion, and metastasis and induce the apoptosis of cancer cells [32,33,34]. Importantly, *miR-101* inhibits tumor proliferation and migration and triggers apoptosis by targeting EZH2, or by connecting to lncRNAs, resulting in dysregulation of each other’s expression. LncRNAs can act as competing endogenous RNAs to *miR-101*. Conversely, *miR-101* has the potential to suppress expression of lncRNAs such as *MALAT1*, *PSMA3-AS1*, *MANCR* and *XIST* [17,32,33,34]. MiR-101 deficiency may also impair negative feedback of proliferation signaling [5,35]. Recent studies have revealed that additional miRNAs, including miR-421, miR-550a-1, and miR-3648, could serve as new prognostic biomarkers in EAC. These miRNAs play a significant role in EAC pathogenesis by suppressing proliferation, invasion, and migration in OE33 cells or by targeting immune-related genes (IRGs) [36,37]

It has been found that in many cancerous tumors, *miR-101* is significantly downregulated compared to healthy tissues [17]. Our research outcomes validate this finding in the context of EAC tumors and positive lymph nodes, areas where there have been limited data on miR-101. Huang SD et al. stated that *miR-101* downregulation exhibits a noteworthy correlation with advanced tumor stage and lymph node metastasis, suggesting its potential involvement in the metastatic processes of ESCCs. Furthermore, this lower expression is linked to an unfavorable prognosis in patients with ESCC [32]. However, miR-101 expression was not significantly correlated with pathological grade in their study [32]. In our analysis, miR-101 expression showed associations with advanced tumor stage, lymph node invasion and pathological grade.

Although statistically insignificant, the patients in our study with low *miR-101* expression also presented a trend towards poorer OS. He XP et al. reported that in gastric cancer, individuals with stage III–IV disease or those with positive lymph nodes exhibited notably reduced miR-101 levels in comparison to patients with stage I–II disease or those with negative lymph nodes [38]. Lower miR-101 levels are linked to augmented tumor size, advanced TNM classification and clinical stage, heightened microvessel and lymphatic density, poor tumor differentiation, as well as compromised overall and disease-free survival [39,40]. Although we observed similar trends, we did not identify a correlation between miR-101 expression and tumor size or DFS in our study. Dong et al. investigated the relevance of *miR-101* in gastric cancer, suggesting that while *miR-101* serves as an independent predictor for the overall survival of GC patients, its expression does not exhibit correlation with the histological tumor type, TNM stage, and tumor size. Their study revealed that patients with low *miR-101* expression experienced a shorter overall survival compared to those with high *miR-101* expression [41]. In our study, *miR-101* expression was not associated with tumor size either. Our patients presented similar survival patterns to the patients in Dong et al.’s study.

In summary, in order to translate our results into clinical practice, we have shown that *MALAT1*, *MANCR* and *miR-101* hold promise as potential prognostic indicators for EAC patients, depicting worse survival and worse tumor staging when dysregulated. Further experimental studies should be performed to investigate whether those molecules are also good candidates for drug targeting.

## 4. Materials and Methods

### 4.1. Patients and Specimens

All patients who were admitted to our department with a diagnosis of EAC between March 2015 and March 2018 were recruited (n = 50). Postoperative pathological status and survival were recorded. A control group comprising 26 healthy individuals was also recruited. Individuals of the control group had negative esophageal biopsies taken during an endoscopy for other reasons, not related to malignant conditions.

The histologic grade and the status of all specimens were classified according to the criteria of the World Health Organization and TNM staging [42,43]. According to GDPR and medical ethics, all samples were obtained after participating patients provided consent. The study was approved by the Ethics Committee of the authors’ institution.

### 4.2. Methodology

All specimens and lymph nodes from patients and controls underwent formalin fixation and paraffin-embedding. The specimens were sectioned into 1–20 μm thick slides, and the slides were then deparaffinized with xylene, rehydrated with graded alcohol solution, and retrieved with citrate buffer.

### 4.3. RNA Extraction and Gene Expression Analysis

Total RNA extraction from all specimens was conducted using NucleoZOL (Macherey-Nagel, Düren, Germany). CDNA synthesis from the total RNA was accomplished utilizing the TAKARA kit (Takara Bio Europe SAS, Saint-Germain-en-Laye, France) according to the manufacturer’s instructions.

Expression levels of *MALAT1*, *MANCR*, *PSMA3-AS1* and *miR-101* were measured via quantitative real-time polymerase chain reaction (qRT-PCR). To perform qRT-PCR, the KAPA SYBR FAST qPCR mix (KAPA BIOSYSTEMS, Cape Town, South Africa) was employed. We used the SYBR Green method to measure expression levels as described in the literature [44]. Additionally, GAPDH was used as a reference gene for MALAT1, MANCR, and PSMA3-AS1, and U6sn was used as a reference gene for miR-101 [16,45,46,47]. Duplicate reactions were conducted for all samples to guarantee reproducibility and gene expression was normalized to the expression of the relevant reference genes. The sequences of the primers used are presented in Supplemental Table S1 [16,45,46,47]. Fold change was calculated as 2−ΔΔCt and is presented as fold regulation. Genes exhibiting downregulation are represented by the negative inverse of fold change and up-regulated genes are represented by the fold change, as previously described [48]. The expression of each of the genes tested was examined in relation to the size of the tumor, grade, T stage, disease-free survival (DFS), overall survival (OS) and infiltration of the lymph nodes (N stage).

Tumors greater than 4.73 cm in diameter comprised the large tumor group, whereas smaller tumors were labeled as small tumors. Regarding T stage, results were grouped as T1 and T2 in one group, and as T3 and T4 in the other group, respectively. Regarding disease grade, results were also grouped as stages 1 and 2 in one group, and as 3 in the other group.

We further examined the OS and DFS between the aforementioned groups with low and high expression of the genes tested, as well as in patients with negative lymph nodes, as this subgroup may represent a field for ongoing cancer research. Survival curves were plotted utilizing the Kaplan–Meier method and compared through the log-rank test.

### 4.4. Statistical Analysis

GraphPad version 3.00 (GraphPad Software, San Diego, CA, USA) was utilized for all statistical analyses. To compare gene expression between cancer and normal tissues, *p*-values were determined by conducting a Student *t*-test on the replicate 2−ΔCt values for each gene within the two groups. A significance level of *p* < 0.05 was deemed as statistically significant for all comparisons.

## 5. Conclusions

The expression patterns and role of long non-coding and micro RNAs in esophageal adenocarcinoma are still not well-defined. In our study, *MALAT1* and *MANCR* were consistently overexpressed, whereas miR-101 was downregulated in EAC. Notably, heightened levels of *MALAT1* and *MANCR*, as well as reduced levels of miR-101, exhibited a strong correlation with lymph node metastasis and invasion, indicating their potential involvement in the progression of EAC tumors. Moreover, the dysregulation of *MALAT1*, *MANCR*, and *miR-101* may have an impact on the staging of EAC tumors, with *miR-101* specifically influencing the characteristics of these tumors. Both *MANCR* and *miR-101* levels hold promise as potential prognostic indicators for EAC patients. Conversely, our findings suggest that PSMA3-AS1 does not exert a significant influence on the course of EAC. To gain a deeper understanding and validate these results, further research studies involving larger cohorts of EAC patients, as well as mechanistic and functional studies, are essential.

### Limitations

Our study has several limitations. Firstly, the sample size was small, which may limit the generalizability of our findings. Secondly, we did not include the administration and type of neoadjuvant and adjuvant chemotherapy in the evaluation of the results, which could have provided additional insights. Thirdly, it is important to note that our study was conducted at a single institution, which may introduce bias and restrict the applicability of the findings to other settings. Furthermore, to gain a better understanding of the mechanisms involved, it is necessary to conduct further in vitro and in vivo experiments. These experiments should aim to determine the extent to which inhibiting these molecules can suppress tumor growth, migratory capacity, and invasive capacity, thereby confirming their potential oncogenic or tumor-suppressive roles.

Finally, we did not investigate the correlation between the expression of the studied molecules and other factors such as age, gender, smoking status, geographical location, and more. Exploring these correlations could provide valuable insights into the broader context of our findings. In summary, while our study has important implications, it is crucial to consider these limitations and conduct additional research to validate and expand upon our findings.

## Figures and Tables

**Table 1:** Patient and control group clinical characteristics.

	Patients (n = 50) (%)	Control Group (n = 26)
Age	67 years	65 years
Male/Female	37/13	14/12
T stage		
T1	5 (10%)	
T2	14 (28%)	
T3	27 (54%)	
T4	4 (8%)	
N Stage		
N0	18 (36%)	
N1	8 (16%)	
N2	11 (22%)	
N3	13 (26%)	
Grade		
1	2 (4%)	
2	27 (54%)	
3	21 (42%)	
Size		
>4.73 cm	27 (54%)	
<4.73 cm	23 (46%)	
Follow-up(months)	3–60	

**Table 2:** Studied molecules’ fold changes between EAC tumors, the cancer-free margin of the specimen, positive lymph nodes (LN+), negative lymph nodes (LN−) and healthy individuals. Triple asterisks (***) denote statistical significance (*p* < 0.05) in comparison to healthy individuals.

	*MALAT1*	*MANCR*	*PSMA3-AS1*	*miR-101*
**Tumor**	1.90 ***	2.58 ***	1.00	−15.95 ***
**Margin**	1.26	1.20	1.01	−2.45
**LN+**	1.97 ***	2.11 ***	1.12	−7.82 ***
**LN−**	1.09	1.12	1.00	−1.02

**Table 3:** Statistical significance (*p* value) in *MALAT1* concentration in various N stages. NS—not significant; S—significant; Τ—trend.

Ν Stage	0	1	2	3
**0**		0.25 (NS)	0.01 (S)	0.06 (T)
**1**			0.51 (NS)	0.96 (NS)
**2**				0.34 (NS)

**Table 4:** Statistical significance (*p* value) in *MANCR* concentration in various N stages. NS—not significant; S—significant; Τ—trend.

Ν Stage	0	1	2	3
**0**		0.45 (NS)	0.034 (S)	0.03 (S)
**1**			0.06 (T)	0.06 (T)
**2**				0.93 (NS)

## Data Availability

Data contained within the article.

## Supplementary Materials

The following supporting information can be downloaded at: https://www.mdpi.com/article/10.3390/ijms25010098/s1.

## References

[1] Yu F.J., Zheng J.J., Dong P.H., Fan X.M. (2015). Long non-coding RNAs and hepatocellular carcinoma. Mol. Clin. Oncol..

[2] Zhang H.M., Yang F.Q., Yan Y., Che J.P., Zheng J.H. (2014). High expression of long non-coding RNA SPRY4-IT1 predicts poor prognosis of clear cell renal cell carcinoma. Int. J. Clin. Exp. Pathol..

[3] Liu Y., Zhang R., Qiu F., Li K., Zhou Y., Shang D., Xu Y. (2015). Construction of a lncRNA-PCG bipartite network and identification of cancer-related lncRNAs: A case study in prostate cancer. Mol. Biosyst..

[4] Hansji H., Leung E.Y., Baguley B.C., Finlay G.J., Askarian-Amiri M.E. (2014). Keeping abreast with long non-coding RNAs in mammary gland development and breast cancer. Front. Genet..

[5] Yu Y., Chen X., Cang S. (2019). Cancer-related long noncoding RNAs show aberrant expression profiles and competing endogenous RNA potential in esophageal adenocarcinoma. Oncol. Lett..

[6] Lu J., Yang J., Ma C., Wang X., Luo J., Ma X., Fu X., Zheng S. (2023). Model construction and risk analysis of the lncRNA genes associated with the prognosis of esophageal adenocarci-noma with immune infiltration. J. Gastrointest. Oncol..

[7] Ravillah D., Kieber-Emmons A.L., Singh S., Keerthy K., Blum A.E., Guda K., BETRNet Consortium (2023). Discovery and Initial Characterization of Long Intergenic Noncoding RNAs Associated with Esophageal Adenocarcinoma. Gastroenterology.

[8] Peng Y., Croce C.M. (2016). The role of MicroRNAs in human cancer. Signal Transduct. Target. Ther..

[9] Syllaios A., Moris D., Karachaliou G.S., Sakellariou S., Karavokyros I., Gazouli M., Schizas D. (2021). Pathways and role of MALAT1 in esophageal and gastric cancer. Oncol. Lett..

[10] Li Santi A., Gorrasi A., Alfieri M., Montuori N., Ragno P. (2018). A novel oncogenic role for urokinase receptor in leukemia cells: Molecular sponge for oncosuppressor mi-croRNAs. Oncotarget.

[11] Yao L., Yan J., Gan L., Huang S., Cheng F., Fang N. (2019). Upregulation of MANCR predicts poor survival in patients with gastric cancer. Oncol. Lett..

[12] Qiu B.Q., Lin X.H., Ye X.D., Huang W., Pei X., Xiong D., Long X., Zhu S.-Q., Lu F., Lin K. (2020). Long non-coding RNA PSMA3-AS1 promotes malignant phenotypes of esophageal cancer by modulating the miR-101/EZH2 axis as a ceRNA. Aging.

[13] Tripathi V., Ellis J.D., Shen Z., Song D.Y., Pan Q., Watt A.T., Freier S.M., Bennett C.F., Sharma A., Bubulya P.A. (2010). The nuclear-retained noncoding RNA MALAT1 regulates alternative splicing by modulating SR splicing factor phosphorylation. Mol. Cell..

[14] Wen S., Zeng M., Li Y., Hu X., Li S., Liang X., Zhu L., Yang S. (2019). Downregulation of MANCR inhibits cancer cell proliferation in mantle cell lymphoma possibly by interacting with RUNX2. Acta Biochim. Biophys. Sin..

[15] Nagasawa M., Tomimatsu K., Terada K., Kondo K., Miyazaki K., Miyazaki M., Motooka D., Okuzaki D., Yoshida T., Kageyama S. (2020). Long non-coding RNA MANCR is a target of BET bromodomain protein BRD4 and plays a critical role in cellular migration and invasion abilities of prostate cancer. Biochem. Biophys. Res. Commun..

[16] Sun D., Li F., Liu L., Yu S., Wang H., Gao X., Liu G., Zhao Y., Qiu G., Jiang X. (2022). PSMA3-AS1 induced by transcription factor PAX5 promotes cholangiocarcinoma proliferation, migration and invasion by sponging miR-376a-3p to up-regulate LAMC1. Aging.

[17] Syllaios A., Sakellariou S., Garmpis N., Sarlani E., Damaskos C., Apostolou K., Kykalos S., Gazouli M., Karavokyros I., Schizas D. (2021). The role of miR-101 in esophageal and gastric cancer. Per. Med..

[18] Klinge C.M. (2018). Non-coding RNAs: Long non-coding RNAs and microRNAs in endocrine-related cancers. Endocr. Relat. Cancer.

[19] Engreitz J.M., Sirokman K., McDonel P., Shishkin A.A., Surka C., Russell P., Grossman S.R., Chow A.Y., Guttman M., Lander E.S. (2014). RNA-RNA interactions enable specific targeting of noncoding RNAs to nascent Pre-mRNAs and chromatin sites. Cell.

[20] Zhang Q.Q., Cui Y.H., Wang Y., Kou W.Z., Cao F., Cao X.J., Miao Z.H., Kang X.H. (2017). Mechanism of long non-coding RNA-metastasis associated lung adenocarcinoma transcript 1 induced invasion and metastasis of esophageal cancer cell EC-109. Zhonghua Zhong Liu Za Zhi.

[21] Wang W., Zhu Y., Li S., Chen X., Jiang G., Shen Z., Qiao Y., Wang L., Zheng P., Zhang Y. (2016). Long noncoding RNA MALAT1 promotes malignant development of esophageal squamous cell carcinoma by targeting β-catenin via Ezh2. Oncotarget.

[22] Zhang Y., Xu Y., Qiu G., Luo Y., Bao Y., Lu J., Wang T., Wang Y. (2023). METTL3 Mediated MA-LAT1 m6A Modification Promotes Proliferation and Metastasis in Osteosarcoma Cells. Molecular Biotechnology.

[23] Yadav A., Biswas T., Praveen A., Ganguly P., Bhattacharyya A., Verma A., Datta D., Ateeq B. (2023). Targeting MALAT1 Augments Sensitivity to PARP Inhibition by Impairing Homologous Recombination in Prostate Cancer. Cancer Res. Commun..

[24] Kang K., Huang Y.H., Li H.P., Guo S.M. (2018). Expression of UCA1 and MALAT1 long-chain non-coding RNAs in esophageal squamous cell carcinoma tissues is predictive of patient prognosis. Arch. Med. Sci..

[25] Li X., Zhao J., Zhang H., Cai J. (2020). Silencing of lncRNA metastasis-associated lung adenocarcinoma transcript 1 inhibits the proliferation and promotes the apoptosis of gastric cancer cells through regulating microRNA-22-3p-mediated ErbB3. OncoTargets Ther..

[26] Hao L., Wu W., Xu Y., Chen Y., Meng C., Yun J., Wang X. (2023). LncRNA-MALAT1: A Key Participant in the Occurrence and Development of Cancer. Molecules.

[27] Li J., Gao J., Tian W., Li Y., Zhang J. (2017). Long non-coding RNA MALAT1 drives gastric cancer progression by regulating HMGB2 modulating the miR-1297. Cancer Cell Int..

[28] Zhang X., Li H., Mao M., Wang X., Zheng J., Yang S. (2019). Mitotically-associated long non-coding RNA promotes cancer cell proliferation in hepatocellular carcinoma by downregulating miR-122a. Oncol. Lett..

[29] Fan J., Wang F. (2021). MANCR drives esophageal carcinoma progression by targeting PDE4D. J. BUON.

[30] Kan L., Yang M., Zhang H. (2023). Long noncoding RNA PSMA3-AS1 functions as a competing endogenous RNA to promote gastric cancer progression by regulating the miR-329-3p/ALDOA axis. Biol. Direct.

[31] Huang T., Chen Y., Zeng Y., Xu C., Huang J., Hu W., Chen X., Fu H. (2021). Long non-coding RNA PSMA3-AS1 promotes glioma progression through modulating the miR-411-3p/HOXA10 pathway. BMC Cancer.

[32] Huang S.D., Yuan Y., Zhuang C.W., Li B.L., Gong D.J., Wang S.G., Zeng Z.-Y., Cheng H.-Z. (2012). MicroRNA-98 and microRNA-214 post-transcriptionally regulate enhancer of zeste homolog 2 and inhibit migration and invasion in human esophageal squamous cell carcinoma. Mol. Cancer.

[33] Lin C., Huang F., Li Q.Z., Zhang Y.J. (2014). miR-101 suppresses tumor proliferation and migration, and induces apoptosis by targeting EZH2 in esophageal cancer cells. Int. J. Clin. Exp. Pathol..

[34] Wang X., Li M., Wang Z., Han S., Tang X., Ge Y., Zhou L., Zhou C., Yuan Q., Yang M. (2015). Silencing of long noncoding RNA MALAT1 by miR-101 and miR-217 inhibits proliferation, migration, and invasion of esophageal squamous cell carcinoma cells. J. Biol. Chem..

[35] Liu N., Yang C., Gao A., Sun M., Lv D. (2022). MiR-101: An Important Regulator of Gene Expression and Tumor Ecosystem. Cancers.

[36] Ji Y., Wang L., Chang G., Yan J., Dai L., Ji Z., Liu J., He M., Xu H., Zhang L. (2023). Mir-421 and mir-550a-1 are potential prognostic markers in esophageal adenocarcinoma. Biol. Direct..

[37] Zhang D., Yin H., Bauer T.L., Rogers M.P., Velotta J.B., Morgan C.T., Du W., Xu P., Qian X. (2021). Development of a novel miR-3648-related gene signature as a prognostic biomarker in esophageal adenocarcinoma. Ann. Transl. Med..

[38] He X.P., Shao Y., Li X.L., Xu W., Chen G.S., Sun H.H., Xu H.-C., Xu X., Tang D., Zheng X.-F. (2012). Downregulation of miR-101 in gastric cancer correlates with cyclooxygenase-2 overexpression and tumor growth. FEBS J..

[39] Liu H.T., Xing A.Y., Chen X., Ma R.R., Wang Y.W., Shi D.B., Zhang H., Li P., Chen H.-F., Li Y.-H. (2015). MicroRNA-27b, microRNA-101 and microRNA-128 inhibit angiogenesis by down-regulating vascular endothelial growth factor C expression in gastric cancers. Oncotarget.

[40] Imamura T., Komatsu S., Ichikawa D., Miyamae M., Okajima W., Ohashi T., Kiuchi J., Nishibeppu K., Kosuga T., Konishi H. (2017). Low plasma levels of miR-101 are associated with tumor progression in gastric cancer. Oncotarget.

[41] Dong X.Q., Zhang Y.H., Shang X.Q., Zeng Y.J. (2019). Effects of miR-101 on the proliferation and apoptosis of gastric mucosal epithelial cells via Nrf2/ARE signaling pathway. Eur. Rev. Med. Pharmacol. Sci..

[42] Nagtegaal I.D., Odze R.D., Klimstra D., Paradis V., Rugge M., Schirmacher P., Washington K.M., Carneiro F., Cree I.A. (2020). The 2019 WHO classification of tumours of the digestive system. Histopathology.

[43] Rice T.W., Patil D.T., Blackstone E.H. (2017). 8th edition AJCC/UICC staging of cancers of the esophagus and esophagogastric junction: Application to clinical practice. Ann. Cardiothorac. Surg..

[44] Wu X., Dai L., Zhang Z., Zheng J., Zhao J. (2020). Overexpression of microRNA-203 can downregulate survivin and function as a potential therapeutic target in papillary thyroid cancer. Oncol. Lett..

[45] Yang H., Liang N., Wang M., Fei Y., Sun J., Li Z., Xu Y., Guo C., Cao Z., Li S. (2017). Long noncoding RNA MALAT-1 is a novel inflammatory regulator in human systemic lupus erythematosus. Oncotarget.

[46] Huang N.S., Lei B.W., Tan L.C., Yu P.C., Shi X., Wang Y., Ji Q.H., Wei W.J., Lu Z.W., Wang Y.L. (2020). Mitotically associated long non-coding RNA is a tumor promoter in anaplastic thyroid cancer. Ann. Transl. Med..

[47] Li M., Tian L., Ren H., Chen X., Wang Y., Ge J., Wu S., Sun Y., Liu M., Xiao H. (2015). MicroRNA-101 is a potential prognostic indicator of laryngeal squamous cell carcinoma and modulates CDK8. J. Transl. Med..

[48] Kambara T., Amatya V.J., Kushitani K., Suzuki R., Fujii Y., Kai Y., Miyata Y., Okada M., Takeshima Y. (2020). SOX6 is a Novel Immunohistochemical Marker for Differential Diagnosis of Epithelioid Mesothelioma from Lung Adenocarcinoma. Am. J. Surg. Pathol..

