# Supplementary material for: The Expression Patterns and Implications of MALAT1, MANCR, PSMA3-AS1 and miR-101 in Esophageal Adenocarcinoma
Source: Int J Mol Sci. 2023 Dec 20;25(1):98. doi: 10.3390/ijms25010098 (PMC10778904; doi:10.3390/ijms25010098)

Supplemental Table S1: Primers used in this study

Molecule names	Sequences (5' to 3')
MALAT1	F-GAATTGCGTCATTTAAAGCCTAGTT<br>R-GTTTCATCCTACCACTCCCAATTAAT
MANCR	F-TCCACTCACCACTCGCTCAC<br>R-CAGGATTAGCACGTTCCAGG
PSMA3-AS1	F-AACAGACCATCAGAAGAGAACA<br>R-GAACAGAAACCAGAGCCATACA
MiR-101	F-GAGGGGTACAGTACTGTGATA<br>R-TGCGTGTCGTGGAGTC

Supplementary Figure S1: Expression of MALAT1 in relation to grouped T-stage. ns: not significant

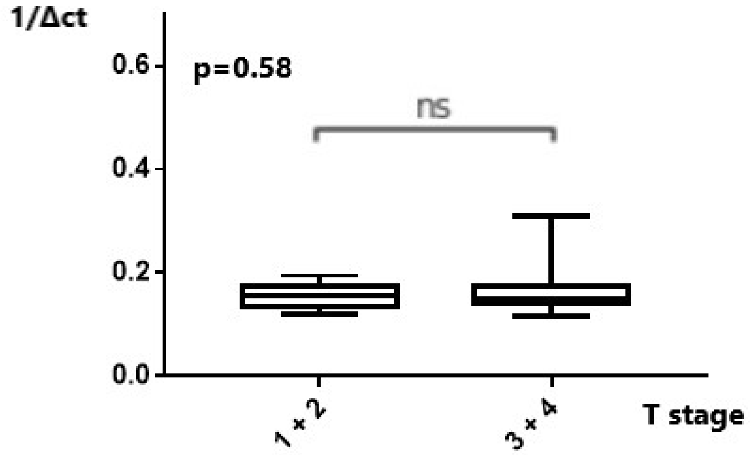

Supplementary Figure S2: Expression of MANCR in relation to grouped T-stage. ns: not significant

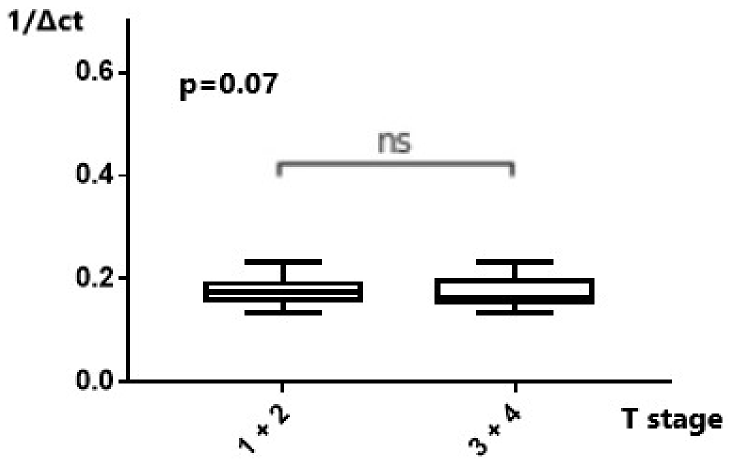

Supplementary Figure S3: Expression of PSMA3-AS1 in relation to grouped T-stage. ns: not significant

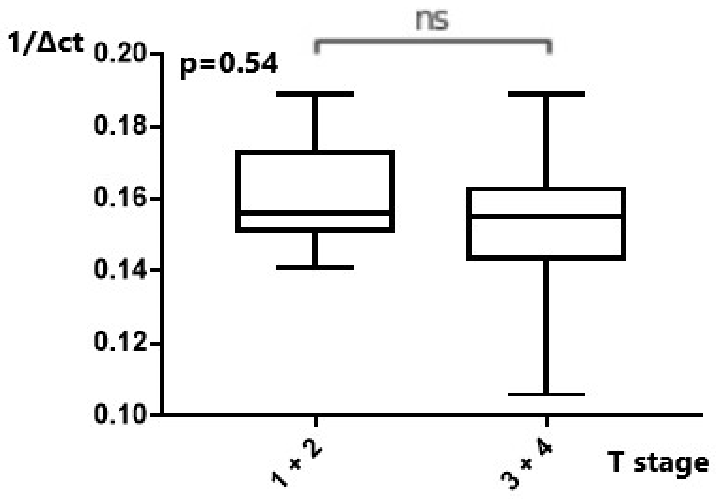

Supplementary Figure S4: Expression of PSMA3-AS1 in relation to N stage. ns: not significant

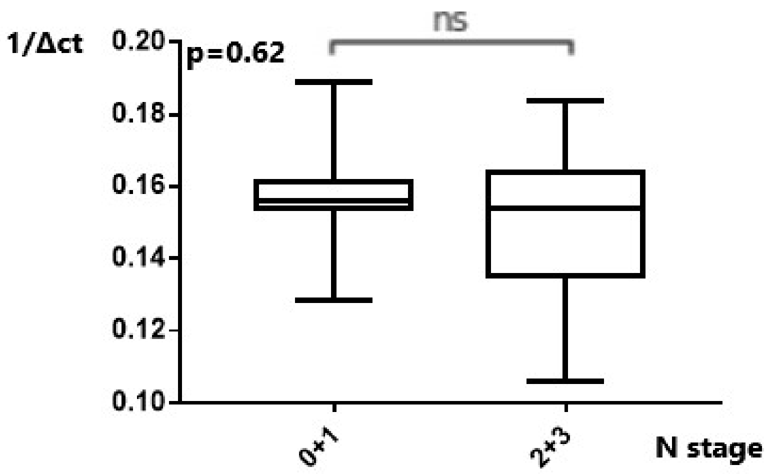

Supplementary Figure S5: Expression of miR-101 in relation to N stage. ns: not significant

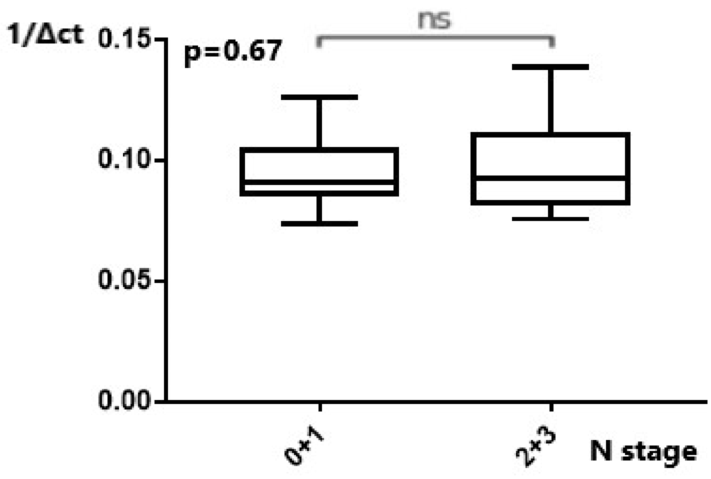

Supplementary Figure S6: Expression of miR-101 in relation to the size of the tumor. ns: not significant

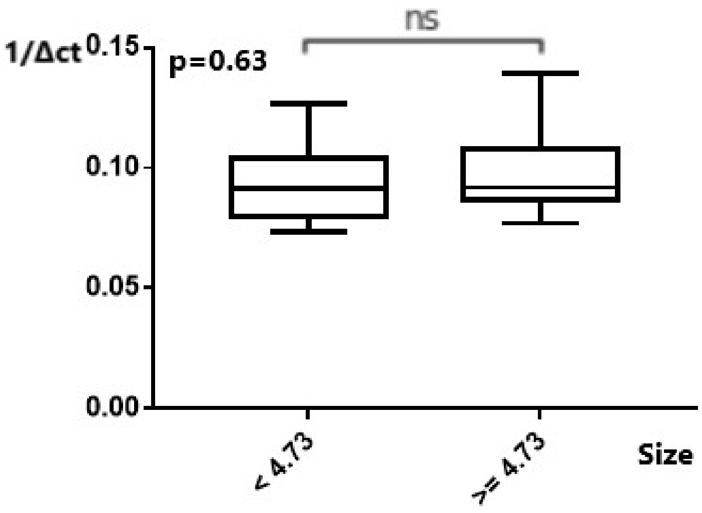

Supplementary Figure S7: Expression of MALAT1 in relation to the size of the tumor. ns: not significant

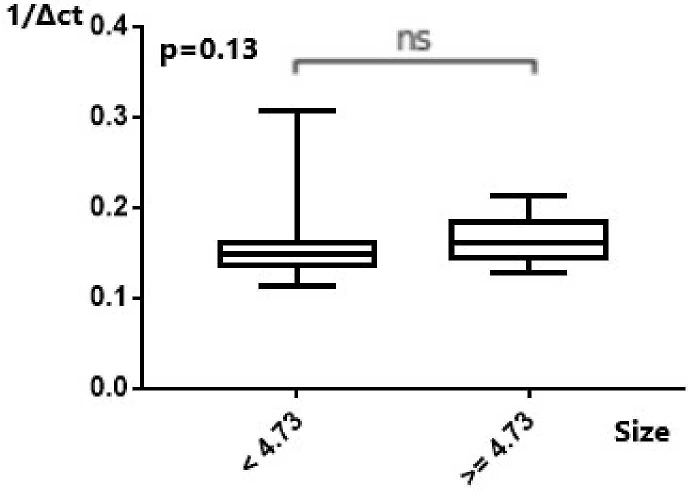

Supplementary Figure S8: Expression of MALAT1 in relation to the grade of the tumor. ns: not significant

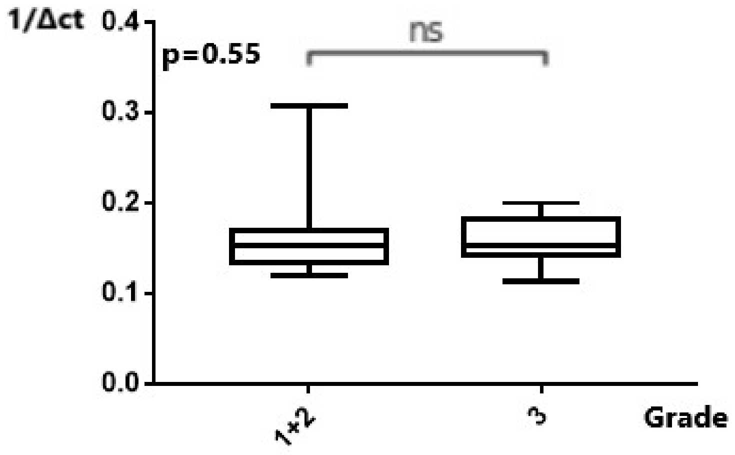

Supplementary Figure S9: Expression of MANCR in relation to grade. ns: not significant

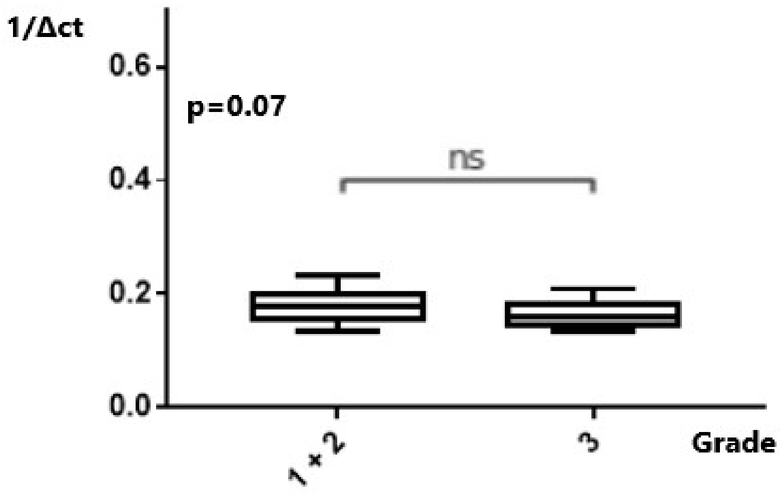

Supplementary Figure S10: Expression of PSMA3-AS1 in relation to grade. ns: not significant

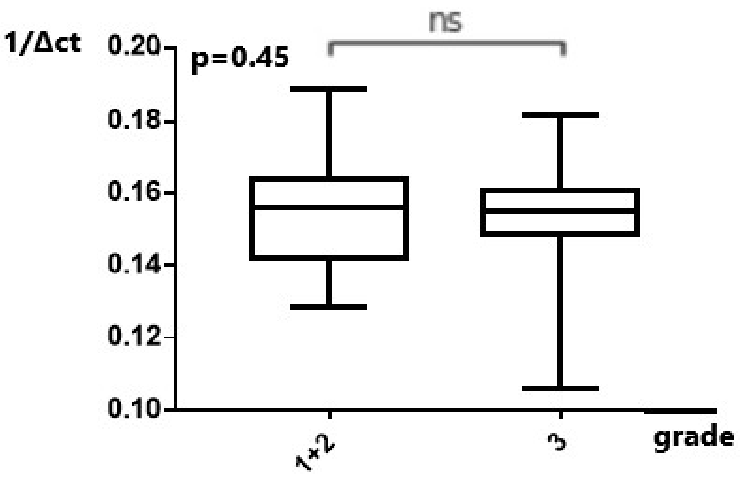

Supplementary Figure S11: MALAT1, overall survival (continuous line: high-MALAT1 expression, intermittent line: low-MALAT1 expression).

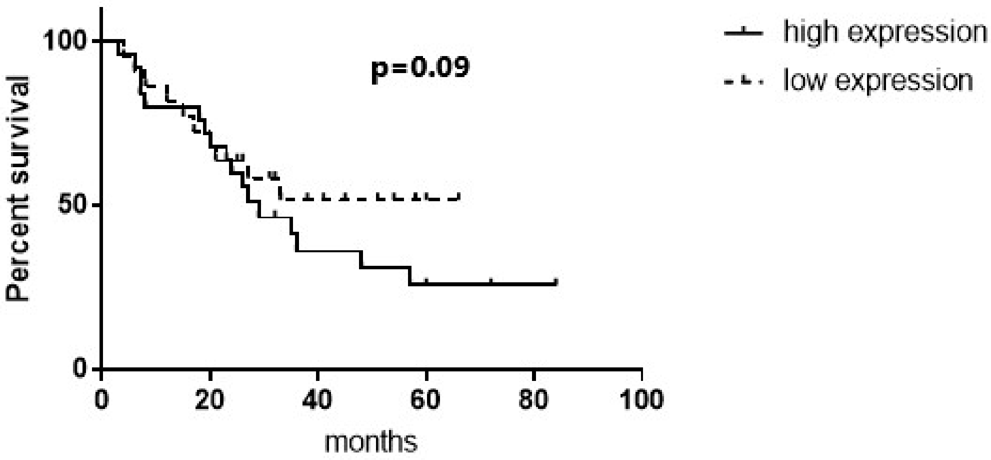

Supplementary Figure S12: MiR-101, overall survival in negative lymph-node patients (continuous line: high-miR-101 expression, intermittent line: low-miR-101 expression).

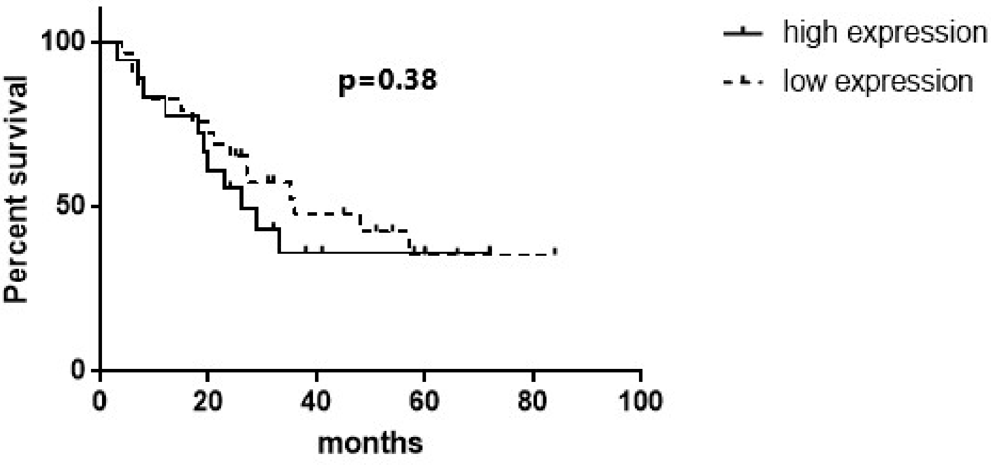

Supplementary Figure S13: MANCR, disease-free survival (continuous line: high-MANCR expression, intermittent line: low-MANCR expression).

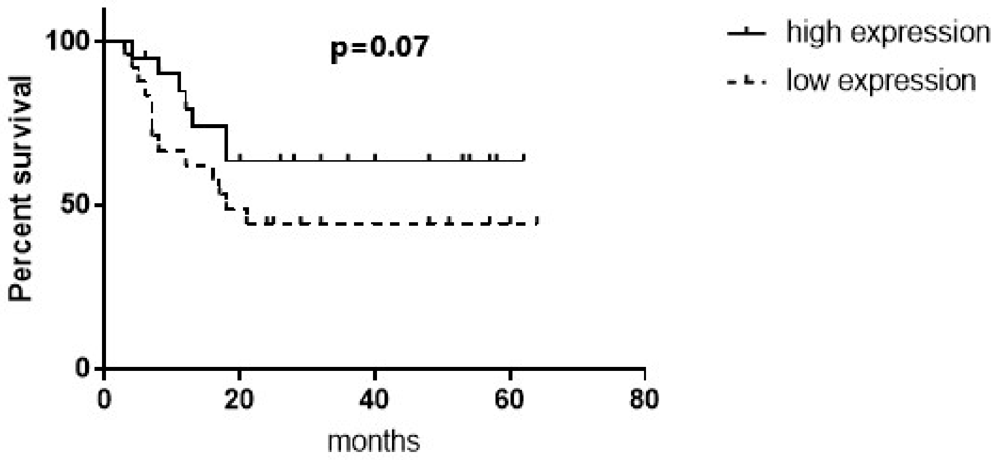

Supplement: Supplementary file 1 [file ijms-25-00098-s001.zip › ijms-2697385-supplementary.pdf]
